# Natural Killer Lytic-Associated Molecule (NKLAM): An E3 Ubiquitin Ligase With an Integral Role in Innate Immunity

**DOI:** 10.3389/fphys.2020.573372

**Published:** 2020-10-29

**Authors:** Donald W. Lawrence, Paul A. Willard, Allyson M. Cochran, Emily C. Matchett, Jacki Kornbluth

**Affiliations:** ^1^Department of Pathology, Saint Louis University School of Medicine, St. Louis, MO, United States; ^2^St. Louis VA Health Care System, St. Louis, MO, United States

**Keywords:** NKLAM, ubiquitin ligase, innate immunity, natural killer, macrophage, phagocytosis, cytotoxicity, RNF19B

## Abstract

Natural Killer Lytic-Associated Molecule (NKLAM), also designated RNF19B, is a unique member of a small family of E3 ubiquitin ligases. This 14-member group of ligases has a characteristic cysteine-rich RING-IBR-RING (RBR) domain that mediates the ubiquitination of multiple substrates. The consequence of substrate ubiquitination varies, depending on the type of ubiquitin linkages formed. The most widely studied effect of ubiquitination of proteins is proteasome-mediated substrate degradation; however, ubiquitination can also alter protein localization and function. Since its discovery in 1999, much has been deciphered about the role of NKLAM in innate immune responses. We have discerned that NKLAM has an integral function in both natural killer (NK) cells and macrophages *in vitro* and *in vivo*. NKLAM expression is required for each of these cell types to mediate maximal killing activity and cytokine production. However, much remains to be determined. In this review, we summarize what has been learned about NKLAM expression, structure and function, and discuss new directions for investigation. We hope that this will stimulate interest in further exploration of NKLAM.

## Introduction to NKLAM

### Discovery of NKLAM

Studies were initiated to identify new genes and gene products associated with cytokine-enhanced natural killer (NK) anti-tumor cytotoxic activity. For these experiments, we used the human NK clone NK3.3, which had been generated previously (Kornbluth et al., 1982). This cell line was cloned from the peripheral blood of a healthy individual, and has all the characteristics of an NK cell. Most importantly, the cytotoxic activity of NK3.3 can be upregulated by cytokine stimulation (Kornbluth and Hoover, 1988). A cDNA library from interferon beta (IFNβ) stimulated NK3.3 cells was made and differential screening was performed to compare expression in unstimulated cells. From this analysis, 56 IFNβ-upregulated genes were identified; 46 were novel at the time. We named one of those novel cDNA clones Natural Killer Lytic-Associated Molecule (NKLAM) (Kozlowski et al., 1999).

Kinetic analysis determined that NKLAM mRNA levels peak 4–6 h after IFNβ stimulation of NK cells; additionally, NKLAM levels are strongly induced by interleukin (IL)-2, peaking 6–12 h after IL-2 stimulation. These expression levels strongly correlate with both IFNβ and IL-2-enhanced NK3.3 anti-tumor cytolytic activity. NKLAM mRNA is short lived, with a half-life of 2.5 h (Kozlowski et al., 1999).

Peripheral blood subsets from healthy donors were isolated and examined for NKLAM mRNA expression. Levels were found to be relatively high in monocytes and upregulated by IFNβ. NKLAM mRNA levels were also high in NK cells and further upregulated by IL-2 and IFNβ. NKLAM mRNA was not found in resting T cells and not induced by IL-2 or the mitogen phytohemagglutinin (PHA). NKLAM was, however, expressed in a CD8^+^ cytotoxic T lymphocyte (CTL) clone and further upregulated by exposure to its target antigen (Kozlowski et al., 1999).

### NKLAM Gene Structure

**FIGURE 1:**
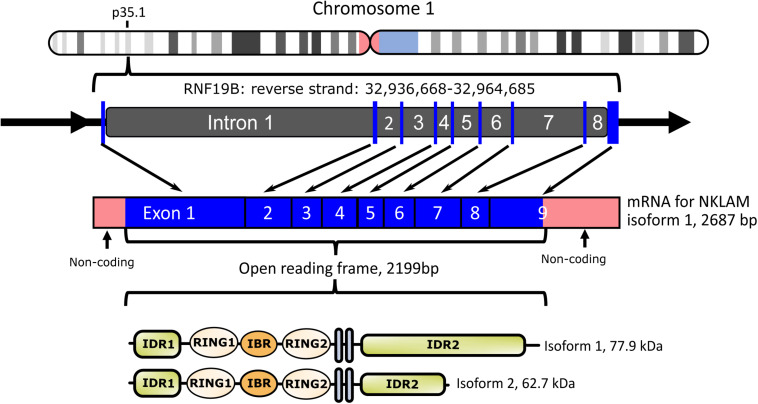
Diagram of human NKLAM (RNF19B) gene organization. NKLAM is encoded by 9 exons (depicted as blue bars) spanning 28 kb in the reverse strand of chromosome 1. Exons 1–3 encode intrinsically disordered region 1 (IDR1) and the three RINGS of the ligase domain. Exons 4 and 5 encode the two transmembrane domains (gray bars), while IDR 2 is encoded across the remaining exons 6–9. Mouse NKLAM, located on chromosome 4, has the same genomic structure. Human NKLAM mRNA isoform 1 encodes a protein with a predicted molecular weight of 77.9 kDa. Alternative splicing of exon 9 results in a shorter mRNA isoform 2 encoding a protein with a predicted molecular weight of 62.7 kDa. Mice only express the long form of NKLAM protein.

We cloned both full-length human and mouse NKLAM (Portis et al., 2000). NKLAM is highly conserved throughout evolution. There is 89% nucleotide and 94% amino acid homology between human and mouse NKLAM. The human NKLAM gene is composed of 9 exons and is found on chromosome 1 (Figure 1). Mouse NKLAM has an almost identical genetic structure of 9 exons and maps to chromosome 4. A major difference is that in humans, there are two forms of NKLAM protein: one is 732 amino acids and the other is 587 amino acids. These arise by alternative splicing in exon 9, resulting in two mRNA transcripts that differ at the 3′ end. Mice (and rats) only produce one mRNA transcript, encoding the longer form of NKLAM protein (Portis et al., 2000). One unresolved question is the functional difference between the two forms of human NKLAM. Both transcripts and proteins are similarly up-regulated and expressed upon activation of human NK cells and macrophages.

### NKLAM Protein Structure and Homology to RING-In Between RING-RING (RBR) Ubiquitin Ligases

When we first described NKLAM, computer analysis predicted it to be a transmembrane protein. It also identified three cysteine-rich clusters, with homology to proteins found in *Caenorhabditis elegans*, mosquitos and in the mouse ovary. This cysteine-rich domain is 99% identical between mouse and human NKLAM. Subsequent cloning and characterization of additional proteins with this signature domain placed NKLAM within the E3 RBR ubiquitin ligase family of proteins. Ubiquitination is one of the most important post-translational modifications, regulating both the stability, function and localization of proteins. Ubiquitin ligases modify proteins by adding either a single ubiquitin or polyubiquitin chains (Swatek and Komander, 2016). Over 800 ubiquitin ligases are known; only 14 have this RBR structure (Smit and Sixma, 2014; Spratt et al., 2014; Lechtenberg et al., 2016; Dove and Klevit, 2017). In addition, only 4 of these are transmembrane proteins like NKLAM. All the RBR E3 ubiquitin ligases are highly conserved and important in cellular physiology. The most studied RBR ligase is Parkin; mutations in Parkin are associated with autosomal recessive juvenile Parkinson’s disease (Kitada et al., 1998). By their ability to control expression of key regulatory proteins involved in cell growth and death signaling, ubiquitin ligases also have a role in development of autoimmunity and cancer (Sun, 2006).

Ubiquitination is a three-step process that involves an E1 activating enzyme that uses ATP and a catalytic cysteine to activate ubiquitin, an E2 conjugating enzyme that accepts the activated ubiquitin from the E1, and an E3 ligase that coordinates the ligation of the E2-bound active ubiquitin onto the target protein (Hochstrasser, 1996). There are three families of E3 ubiquitin ligases: HECT-type, RING-type, and RBR-type ligases. HECT ligases contain the HECT (homologous to the E6-AP carboxyl terminus) domain which provides a catalytic cysteine to accept active ubiquitin from the E2 and form an intermediate E3-ubiquitin prior to ligation onto the substrate (Weber et al., 2019). This frees the E2 and enables the HECT E3 ligase to dictate whether the ubiquitin is added singly to a target substrate or ligated to create a ubiquitin chain. RING ligases are identified by the presence of a RING (Really Interesting New Gene) domain of the canonical C3HC4 structure. RING ligases do not have a catalytic cysteine, but function as a scaffold, facilitating the direct transfer of the active ubiquitin from the E2 to the substrate (Metzger et al., 2014). It is, therefore, the E2 that determines the ubiquitin linkage that is created. RBR ligases are composed of a C3HC4 RING domain (RING1), identical to RING-type ligases, followed by an In-Between RING (IBR), and another RING (RING2) domain. RING2 contains a conserved catalytic cysteine for accepting active ubiquitin to form an intermediate E3-ubiquitin, similar to HECT-type ligases (Spratt et al., 2014; Walden and Rittinger, 2018). RBR ligases control ubiquitin linkage type in a manner similar to HECT ligases.

The conserved catalytic cysteine in the RING2 domain of RBR E3 ubiquitin ligases, including Parkin and Dorfin (RNF19A), is considered a defining feature of this ligase family. NKLAM also has a comparable cysteine (C302) that we predict is critical for its ligase activity (Lechtenberg et al., 2016; Dove and Klevit, 2017).

RING-IBR-RING ubiquitin ligases also have preference for the E2s that they interact with. We identified the ubiquitin conjugating enzymes UbcH7 and UbcH8 interacting with NKLAM. There is a level of E2 specificity; UbcH10 is unable to bind NKLAM (Fortier and Kornbluth, 2006). Upon stimulation of NK cells with IFNβ, both NKLAM and UbcH8 are upregulated and can be co-immunoprecipitated. These ubiquitin conjugates bind to the RING domain of NKLAM. Most E2-conjugating enzymes transfer ubiquitins onto both lysine and cysteine residues and therefore function with multiple types of ubiquitin ligases. However, UbcH7 is unique in being strictly cysteine-reactive and works predominantly with RBR E3 ligases like NKLAM (Martino et al., 2018).

**FIGURE 2:**
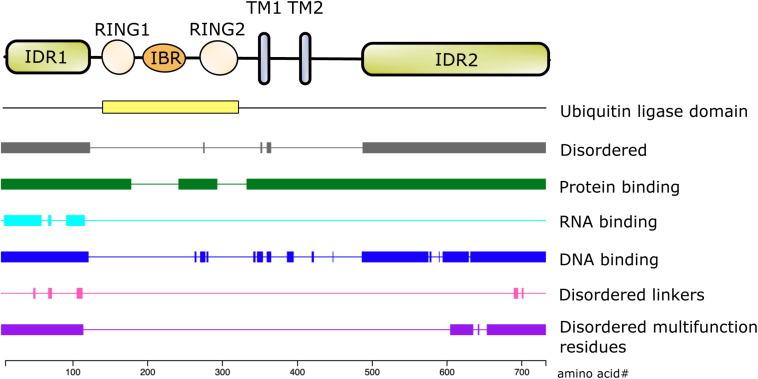
Structure/function of NKLAM protein. The RING1-IBR-RING2 (RBR) domain has the ubiquitin ligase activity. RING2 contains the highly conserved, active site catalytic cysteine (C302), which is the defining feature of this family of RBR ligases. In addition to the two transmembrane (TM) domains, the N- and C-terminal ends of NKLAM are intrinsically disordered regions (IDR). Bioinformatic analysis using the DEPICTER server indicate the possible roles of the IDR in protein, DNA, and RNA binding.

The first 100 amino acids of NKLAM consist primarily of alanine, arginine, glutamate, glycine, and proline. Bioinformatic analysis using the DEPICTER server identifies this amino acid stretch as an intrinsically disordered region (IDR) of the protein (Barik et al., 2020; Figure 2). This combination and arrangement of amino acids is predicted to give NKLAM the capability to bind RNA, DNA, and protein. Following the N-terminal IDR is the RBR ligase domain of NKLAM. There are two transmembrane domains that anchor the protein, and a second larger IDR at the C-terminal end of NKLAM that is also predicted to interact with DNA and proteins.

Intrinsically disordered regions are typically rich in charged or polar residues, enabling interactions with proteins or nucleic acids (Wright and Dyson, 2015; Dyson, 2016). These regions are often flexible enough to either conform to the surface of an interacting protein, thereby stabilizing the interaction, or to adopt a different conformation to allow interactions with additional ligands (Dunker et al., 2005). Post-translational modifications of these regions can induce changes in conformation, thereby changing the accessibility of binding sites or altering the activity of the protein (Darling and Uversky, 2018; Owen and Shewmaker, 2019).

### Regulation of NKLAM Expression

Natural killer lytic-associated molecule is expressed by a variety of hematopoietic cells, including NK cells, CD8^+^ cytotoxic T cells and murine bone marrow-derived macrophages (BMDM). Our earliest studies of NKLAM demonstrated its expression in freshly isolated human peripheral blood monocytes and upregulation by IFNβ. Similar results were obtained using macrophages from the spleen and peritoneal cavity of mice (Portis et al., 2000). There is limited experimental data on NKLAM expression in non-immune cells; however, NKLAM was found to be associated with ppp1cc in testes (Fardilha et al., 2011). Our laboratory found that NKLAM is expressed in mouse tracheal epithelial cells treated with IFNγ (Lawrence et al., 2019). The experimental data on NKLAM expression is largely derived from NK cell and macrophage studies.

Natural killer lytic-associated molecule expression is induced by both IFNγ and IFNβ; thus, NKLAM is an interferon-stimulated gene (ISG). Resting NK cells have low levels of NKLAM; treatment with IFNβ rapidly induces transcription and translation of NKLAM, where it localizes to lytic granule membranes (Kozlowski et al., 1999). In addition to IFNβ, IL-2, IL-12, IL-15, and IL-21 also promote transcription of NKLAM in NK cells. Similarly, under baseline conditions, macrophages express minimal amounts of NKLAM. Treatment with IFNγ induces NKLAM expression in a time dependent manner with maximal levels reached at 12 h (Portis et al., 2000; Lawrence and Kornbluth, 2012). Using transcription factor binding site prediction software PROMO^1^, the promoter region of NKLAM contains a binding site for STAT1. The signal transduction pathway for IFNγ, a type II interferon, results in STAT1 binding a gamma-activated sequence (GAS) within an ISG promoter region. Additionally, there are STAT4 and STAT5 binding sites in the NKLAM promoter. STAT4 and STAT5 are involved in signal transduction from various cytokine receptors for IL-2, IL-12, and IL-15. The NKLAM promoter also contains binding sites for members of the interferon-regulatory factor (IRF) family of transcription factors. IRF proteins are regulators of the type I interferon system. The expression of NKLAM in mouse immune cells is the same as seen in human cells and its regulation is also mediated by the same activation signals.

Natural killer lytic-associated molecule expression can also be induced by Toll-like receptor (TLR) agonists. Mouse macrophage cell lines RAW 264.7 and J774A.1 were stimulated with TLR4 agonist lipopolysaccharide (LPS), the combination of LPS plus IFNγ, *Escherichia coli*, or *Staphylococcus aureus* and NKLAM protein expression was assessed over time. In both RAW 264.7 and J774 cells, peak NKLAM expression is seen 16 h after stimulation with all treatments (Lawrence and Kornbluth, 2012). Stimulation with LPS plus IFNγ leads to the largest increase in NKLAM levels (Portis et al., 2000). Poly (I:C), a mimetic of double stranded RNA and a TLR3 agonist, was also found to induce NKLAM expression in BMDM (Lawrence et al., 2019). TLR stimulation culminates in the activation of transcription factors NFκB, IRFs, and MAP kinases to regulate gene expression (Kawasaki and Kawai, 2014). In addition to binding sites for IRF-1 and IRF-2, the NKLAM promoter contains binding sites for NFκB proteins p50 and p65/RelA.

## NKLAM Function

### NKLAM Function in Cytotoxic Cells

Natural killer lytic-associated molecule mRNA expression strongly correlates with cytotoxic activity. To determine whether NKLAM is necessary for NK killing, NK cells were treated with NKLAM antisense (AS) oligonucleotides (ODN) or control ODN. There is a significant and specific downregulation of NKLAM expression and cytotoxic function of NK cells after treatment with NKLAM AS ODN. Granule exocytosis-mediated killing is diminished after NKLAM AS ODN treatment; however, Fas-mediated killing appears to be NKLAM-independent. In 4 h killing assays, the cytotoxic activity of CTL against its specific target is reduced by 60% after treatment with NKLAM AS ODN (Kozlowski et al., 1999). This suggests that NKLAM function is associated with killing mediated by lymphocyte granule exocytosis.

We generated a panel of monoclonal antibodies to NKLAM. Resting peripheral blood NK cells express little to no NKLAM; upon cytokine activation, NKLAM protein levels increase. Subcellular fractionation experiments localized NKLAM to the membranes of the cytolytic granules in NK cells. Unlike other granule proteins, NKLAM is not pre-formed; it is rapidly transcribed, translated and embedded in granule membranes upon NK activation (Kozlowski et al., 1999).

To further investigate the function of NKLAM in NK cytotoxic activity, we generated NKLAM-deficient (NKLAM^–/–^) mice (Hoover et al., 2009). This was accomplished by genomic deletion of exons 2–5, which removes the 2nd and 3rd RING domains and both transmembrane domains. These mice are completely NKLAM deficient. By both RT-PCR and protein analysis, there is no detectable full length or fragments of NKLAM in these mice. Mice were backcrossed to C57BL/6 mice for 11 generations. NKLAM^–/–^ mice appear normal and are fertile. The lymphoid and myeloid subpopulations in the spleen are comparable to wild type (WT) mice in numbers and distribution. They also have normal numbers of NK cells in the spleen; these NK cells have similar amounts of cytotoxic proteins perforin and granzyme B in their granules as WT NK cells. Granule release is comparable in both NKLAM^–/–^ and WT NK cells. However, NKLAM^–/–^ NK cells have 60% less tumor killing activity *in vitro* and secrete less IFNγ after target or cytokine stimulation (Hoover et al., 2009).

### NKLAM-Mediated Ubiquitination of Uridine-Cytidine Kinase Like-1 (UCKL-1)

We employed the yeast-two-hybrid system to identify potential substrate proteins that bind to and are ubiquitinated by NKLAM. The RING domain of NKLAM was used as bait to trap binding proteins from a human spleen cell cDNA library in yeast. Using high stringency binding conditions, we identified uridine-cytidine kinase-like 1 (UCKL-1, previously called URKL-1) interacting with NKLAM. Co-transfection studies in HEK293 cells confirmed the interaction between NKLAM and UCKL-1, leading to UCKL-1 ubiquitination and degradation (Fortier and Kornbluth, 2006). NKLAM constructs containing one or more of the RING domains were designed and analyzed for their ability to bind UCKL-1 and ubiquitin conjugates. We demonstrated that the entire RBR domain, without the N-terminal and C-terminal regions of NKLAM, is capable of binding UCKL-1, and ubiquitin conjugates UbcH7 and UbcH8. Although UCKL-1 co-immunoprecipitates with multiple combinations of two of the three RING domains, it is maximally ubiquitinated and degraded by full length NKLAM or the entire RBR domain (Fortier and Kornbluth, 2006). Since the highly conserved catalytic cysteine (C302) in the RING2 domain is considered required for its ubiquitin ligase activity, we generated NKLAM constructs with a cysteine to alanine (C-A) mutation of C302 (C302A). A comparison of WT and mutant NKLAM will determine the role of ubiquitination in NKLAM functions.

Uridine kinases are involved in the pyrimidine salvage pathway. UCKL-1 is often upregulated in cancers and is proposed as a biomarker for several cancer types (Geiger et al., 2012; Cheng et al., 2014). Since uridine kinase expression is associated with cancer growth, we hypothesized that upon NK-tumor cell interaction, during the process of granule exocytosis, NKLAM may be able to interact with tumor-associated proteins, like UCKL-1. This would result in loss of UCKL-1 expression in the tumor cell, thereby impacting its survival.

To model the potential effect of NKLAM on reduction of UCKL-1 expression in tumor cells, we performed siRNA experiments. Downregulation of UCKL-1 in tumor cells by siRNA slows their proliferation, induces apoptosis and enhances their susceptibility to NK-mediated lysis (Ambrose and Kornbluth, 2009). Conversely, over-expression of UCKL-1 protects tumor cells from NK killing and enhances tumor survival *in vitro* and *in vivo* (Gullickson et al., 2016). These data suggest a model where, upon NK-tumor interaction and release of lytic granules, NKLAM enters the tumor cell, ubiquitinates and degrades UCKL-1, thereby promoting cell death.

**FIGURE 3:**
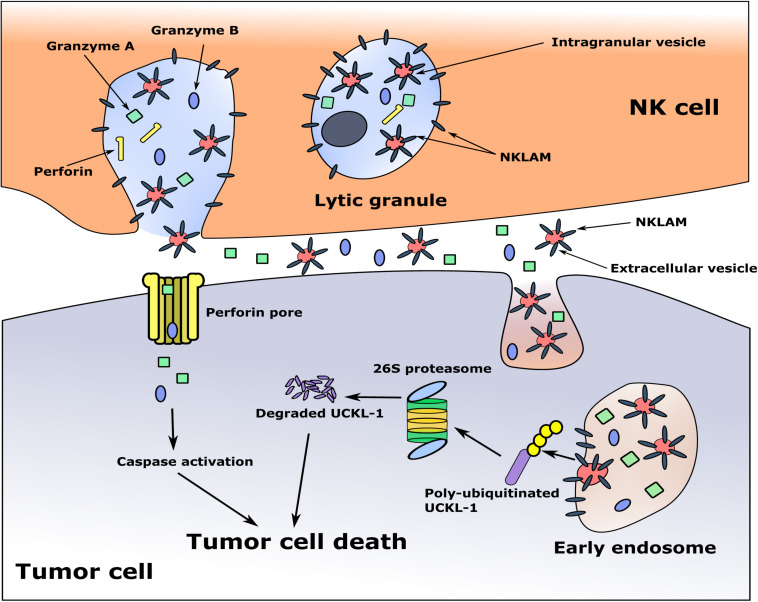
Proposed model of NKLAM function in NK cells. The cytolytic granules of resting NK cells contain little to no NKLAM. Upon cytokine exposure or target stimulation, activated NK cells rapidly transcribe and translate NKLAM, which is embedded in cytolytic granule membranes. Smaller, intragranular vesicles also have NKLAM in their membranes. NK-tumor interaction releases granule contents (perforin, granzymes) into the tumor cell at the immunological synapse. Intragranular vesicles are also released; these now called extracellular vesicles (EVs) contain NKLAM, as well as perforin and granzymes, and enter the tumor cell by endocytosis. Granzymes induce caspase activation; NKLAM ubiquitinates and degrades the tumor survival protein UCKL-1. This combination of perforin, granzymes, and NKLAM ensures death of the tumor cell. Red circles: EVs; gray bars: NKLAM; green squares, blue ovals: cytolytic proteins granzymes A and B; yellow bars: perforin; purple bar: UCKL-1; yellow circles: ubiquitin.

The cytotoxic granules in NK cells are heterogeneous, varying in size and in the amount of electron-dense material in their core. They are called hybrid organelles, with the properties of both lysosomes and secretory granules. This heterogeneity may reflect a continuum of maturation (Burkhardt et al., 1990; De Saint Basile et al., 2010). Some of the granules contain multi-vesicular bodies, where smaller membrane-bound intragranular vesicles, exosomes, are generated. It has been demonstrated that exosomes derived from NK cells contain the cytolytic molecules perforin, granzymes A and B and granulysin, as well as the membrane proteins FasL and CD63, and have anti-tumor cytotoxic activity (Lugini et al., 2012; Jong et al., 2017). During NK-tumor cell interaction and degranulation, exosomes and other extracellular vesicles (EVs) are released, and may participate in tumor killing (Wu et al., 2019). This would provide a mechanism for delivery of the granule membrane protein NKLAM from the NK cell to the target cell, where it would ubiquitinate and degrade UCKL-1, ultimately resulting in cell death. A model of tumor killing is depicted in Figure 3.

### Role of NKLAM in Anti-Tumor Activity *in vivo*

The B16 melanoma model of experimental lung metastasis was used to evaluate NK function in NKLAM^–/–^ mice *in vivo*. This model is widely used in studies of NK function *in vivo* because B16 melanoma cells express low amounts of MHC class I molecules, are poorly immunogenic so they do not generate a significant adaptive immune response, and are readily killed by NK cells (Hanna and Burton, 1981; Gorelik et al., 1982; Warner and Dennert, 1982). NKLAM^–/–^ and WT mice were injected intravenously with B16 melanoma cells; tumor colonies in the lungs were counted 15 days later. NKLAM^–/–^ mice have substantially higher numbers and larger lung melanoma nodules than WT mice. These results indicate the importance of NKLAM in controlling tumor metastasis, likely by enhancing NK anti-tumor function (Hoover et al., 2009).

The role of NKLAM in NK-mediated tumor immunity *in vivo* was further investigated by employing additional tumor models to compare tumor development, progression and metastasis in NKLAM^–/–^ and WT mice. We injected mice with RMA-S, a mouse T cell lymphoma, and well-characterized NK-susceptible hematopoietic tumor. These cells are MHC class I negative and are killed exclusively by NK cells (Kim et al., 2000; Cerwenka et al., 2001; Diefenbach et al., 2001). Using a sensitive real-time quantitative PCR assay for tumor burden, we found greater dissemination of RMA-S tumor cells to the lungs, lymph nodes, bone marrow and blood of NKLAM^–/–^ mice compared to WT mice (Hoover et al., 2012). These results indicate that NKLAM^–/–^ mice are less capable of controlling lymphoma dissemination than WT mice.

The potential role of NKLAM in controlling breast cancer growth and metastasis was evaluated by injecting syngeneic EO771 breast cancer cells into the mammary fat pads of NKLAM^–/–^ and WT mice. EO771 is poorly immunogenic, incapable of generating a primary cytotoxic T cell response *in vivo.* It is also estrogen receptor positive, highly aggressive, and prone to metastasis (Ewens et al., 2005; Zhou et al., 2005; Gu et al., 2009). Primary tumor growth is similar between NKLAM^–/–^ and WT mice. However, there are much higher levels of disseminated tumor cells in the bloodstream and more metastatic EO771 breast cancer cells in the lungs of NKLAM^–/–^ than WT mice (Hoover et al., 2012). These results suggest that NKLAM-expressing NK cells play a key role in controlling tumor dissemination and metastasis *in vivo*. Reversal of these effects by reconstitution of NKLAM^–/–^ mice with WT bone marrow would confirm the role of NKLAM^+^ hematopoietic cells in anti-tumor immunity; adoptive transfer of WT NK cells into NKLAM^–/–^ mice would confirm the role of NKLAM^+^ NK cells in controlling tumor spread.

### NKLAM Function in Macrophages

Macrophages are an important cellular component of the innate and adaptive immune systems and represent a first line of defense against invading pathogens. Macrophages employ oxidative and non-oxidative killing mechanisms to rid the host of pathogens. Since NKLAM is highly expressed in macrophages, we performed macrophage bacterial killing studies to determine whether NKLAM plays a role in macrophage bactericidal activity.

Transient transfection of J774 cells with NKLAM results in increased intracellular killing of *E. coli* compared with control-transfected cells. These results suggest that NKLAM enhances macrophage-mediated bacterial degradation. These data were confirmed in experiments using WT and NKLAM^–/–^ BMDM and peritoneal macrophages isolated from WT and NKLAM^–/–^ mice; WT macrophages have greater bacterial killing activity than NKLAM^–/–^ macrophages (Lawrence and Kornbluth, 2012).

We evaluated each of the steps associated with macrophage bactericidal activity. We measured phagocytosis of fluorescently labeled *E. coli* by flow cytometry and found no significant difference in uptake of bacteria between WT and NKLAM^–/–^ macrophages. We also assayed pH reduction during phagosome maturation by incubating macrophages with *E. coli* labeled with a pH sensitive fluorescent dye (pHrodo). There were no significant differences in phagosome pH between WT and NKLAM^–/–^ macrophages. Additionally, cleavage of cathepsin D, that occurs during phagosome maturation, is also similar between WT and NKLAM^–/–^ macrophages (Lawrence and Kornbluth, 2012).

Phagosome maturation is a highly dynamic process. The number of proteins associated with the phagosome range from hundreds (Garin et al., 2001) to thousands (Trost et al., 2009). These proteins are involved in phagosome trafficking, protein degradation, phagosome acidification, and antigen presentation (Kinchen and Ravichandran, 2008). We isolated phagosomes from WT and NKLAM^–/–^ macrophages and found that NKLAM is a component of macrophage phagosomes and co-localizes with phagosome proteins EEA-1 and LAMP-1 (Lawrence and Kornbluth, 2012). Using immunofluorescence, we localized NKLAM to the phagosome membrane surrounding ingested fluorescently labeled bacteria (Lawrence and Kornbluth, 2012). NKLAM expression in the phagosome is maximal at ∼35 min post-ingestion and this correlates with increased ubiquitination of phagosome proteins; however, the identity of NKLAM phagosome targets is unknown. Ubiquitinated proteins on the phagosome membrane have been shown to interact with ESCRT (endosomal sorting complex required for transport) machinery and are necessary for protein sorting and phagosome maturation (Migliano and Teis, 2018). A recent study by Dean et al. (2019) suggests that post-translational modification of phagosome proteins (e.g., phosphorylation and ubiquitination) may transform the phagosome into a subcellular signaling platform. In support of this concept, a mass spectrometry (MS)-based analysis of macrophage phagosome proteins revealed the presence of ubiquitin conjugation machinery including E1, E2, and E3 enzymes (Guo et al., 2015).

**FIGURE 4:**
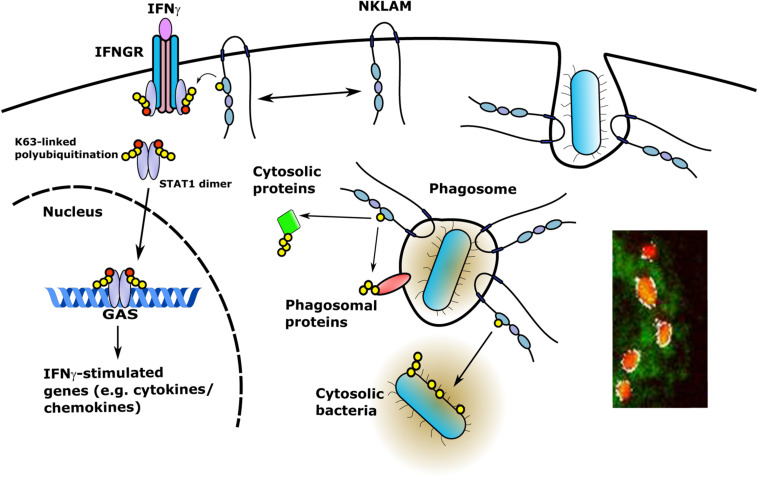
NKLAM in macrophage immune function. Upon exposure to IFNγ, STAT1 is phosphorylated and NKLAM is transiently associated with the IFNγ-IFNGR complex at the plasma membrane. In this location, NKLAM is in close proximity to STAT1, which would allow NKLAM to ubiquitinate STAT1 or other proteins to promote STAT1 transcriptional activity. After ingestion of bacterial targets, NKLAM is localized to the phagosome membrane with the catalytic RING2 domain and C-terminal tail facing the cytoplasm. This would provide NKLAM with access to phagosome membrane proteins and cytosolic target proteins, as well as bacteria that escape the phagosome. Red circles; phosphotyrosine, yellow circles; ubiquitin. Immunomicrograph insert: Macrophage phagosomes after ingestion of fluorescent-labeled *E. coli* (red). NKLAM staining: green; Image J-defined co-localization of *E. coli* and NKLAM: white. Adapted from Lawrence, D.W., and Kornbluth, J. E3 ubiquitin ligase NKLAM is a macrophage phagosome protein and plays a role in bacterial killing. Copyright 2012, with permission from Elsevier and Lawrence D.W., and Kornbluth, J. E3 ubiquitin ligase NKLAM ubiquitinates STAT1 and positively regulates STAT1-mediated transcriptional activity. Copyright 2016, with permission from Elsevier.

Since NKLAM is a membrane-bound protein, investigation of the orientation of the catalytic RING domain will determine whether NKLAM has access to phagosome cargo or cytosolic targets. If NKLAM is embedded in the phagosome membrane with its catalytic RING domains and C-terminal tail orientated into the cytoplasm, it would have access to phagosome membrane and cytoplasmic targets (Figure 4). In such an orientation, NKLAM would have access to bacteria that have evolved phagosomal escape mechanisms (e.g., *Mycobacterium tuberculosis* and *Listeria monocytogenes*). Another RBR ligase, Parkin, has been shown to ubiquitinate cytosolic *M. tuberculosis*, reducing its replication via destruction in the autophagolysosome (Manzanillo et al., 2013). Identification of NKLAM targets will be critical for determining the role of NKLAM in macrophage bactericidal function.

The processes of phagocytosis and autophagy have overlapping roles and mechanisms. LC3-associated phagocytosis (LAP) is a process in which some components of the autophagy pathway are recruited to the phagosome to lipidate LC3 molecules on a single membrane. Despite some overlap, LAP and canonical autophagy are distinct at the molecular, cellular, and functional levels. After phagocytic cargo uptake and phagosome formation, the class 3 PI-3-kinase complex (PI3KC3) is the first to be recruited to the membrane (Heckmann et al., 2017; Herb et al., 2020). This process is shared between LAP and canonical autophagy. Affinity purification MS has identified numerous proteins that interact with NKLAM (Huttlin et al., 2015, 2017). The BioGRID^2^ database lists 56 interacting proteins; of these, three are members of the PI-3 kinase complex. This would place NKLAM not only in the phagosome membrane, but also in the membrane of the LAPosome. Our recent studies using a Sendai virus pneumonia model show that the lack of NKLAM negatively affects not only the conversion of LC3I to LC3II but the overall expression of LC3 (Lawrence et al., 2019). This observation implicates NKLAM as a potential regulator of LAP through its regulation of LC3 protein expression. Further studies are needed to determine whether NKLAM acts locally at the level of the LAPosome or more systemically at the transcriptional level.

### NKLAM Regulation of Immune-Associated Transcription Factor Activity

Transcription factors that are crucial to the immune response are proteins of the STAT and NFκB families. Studies from our laboratory have shown that NKLAM plays an important role in regulating the activity of key members of both groups of transcription factors.

Using a transfection-based approach in HEK293 cells, we observed that NKLAM and STAT1 are associated in a protein complex (Lawrence and Kornbluth, 2016). Similar results were obtained using RAW 264.7 macrophages. *In vitro*, NKLAM is transiently localized to the interferon gamma receptor (IFNGR) during BMDM stimulation with IFNγ (Lawrence and Kornbluth, 2016). STAT1 immunoprecipitated from WT and NKLAM^–/–^ BMDM during IFNγ stimulation shows evidence of increased transient K63-linked polyubiquitination in WT but not in NKLAM^–/–^ cells. Total STAT1 levels are not significantly altered during the time course, suggesting that polyubiquitination does not induce large-scale STAT1 degradation (Lawrence and Kornbluth, 2016).

The observation that NKLAM associates with STAT1 and has a positive effect on its K63-linked polyubiquitination prompted us to determine if STAT1 DNA binding and/or transcriptional activity are affected by NKLAM. To that end, we performed oligonucleotide pull down assays. Our results demonstrated that the lack of NKLAM negatively affects STAT1 binding to an oligonucleotide containing a GAS (Lawrence and Kornbluth, 2016). In support of this observation, transfection studies using a GAS luciferase reporter plasmid demonstrated that STAT1-mediated transcriptional activity is lower in NKLAM^–/–^ than in WT cells (Lawrence and Kornbluth, 2016). Precisely which lysines of STAT1 serve as potential NKLAM ubiquitination targets and how ubiquitination affects STAT1 activity remain to be determined. Interestingly, a study by Huntelmann et al. (2014) determined that lysine 567 in STAT1 is required for GAS recognition; however, it has not been determined if that particular lysine is ubiquitinated. Recently, Guo et al. (2020) demonstrated that E3 ligase RNF220 mediated K63-linked ubiquitination of STAT1 at lysine 110 promotes the interaction between STAT1 and JAK1. Similar to our studies, the authors showed that RNF220 expression was induced by IFNγ signaling and importantly, K63-linked ubiquitination of STAT1 promoted cytokine induction (Guo et al., 2020). Additionally, others have shown that K63-linked poly-ubiquitination of transcription factors is a positive regulator of transcriptional activity (Adhikary et al., 2005; Yao et al., 2018).

STAT1 phosphorylation precedes translocation into the nucleus and transcriptional activation. NKLAM affects the phosphorylation state of both STAT1 and STAT3 during active pulmonary infection. In a model of bacterial pneumonia, STAT1 and STAT3 phosphorylation in the lungs of mice infected with *Streptococcus pneumoniae* is significantly lower in NKLAM^–/–^ mice than in WT mice (Lawrence and Kornbluth, 2018). In support of this novel observation, we observed that phosphatase activity is significantly higher in NKLAM^–/–^ mouse lungs infected with *S. pneumoniae* (Lawrence and Kornbluth, 2018). In a parallel study, using a model of viral pneumonia, STAT1 phosphorylation is also lower in lungs from NKLAM^–/–^ mice than from WT mice infected with Sendai virus (SeV) (Lawrence et al., 2019).

The NFκB pathway is considered the prototypical proinflammatory signaling cascade. NFκB regulates the expression of proinflammatory cytokines and chemokines crucial to the immune response. We found that nuclear translocation of the NFκB protein p65 is significantly delayed in NKLAM^–/–^ BMDM compared to WT macrophages after stimulation with LPS (Lawrence et al., 2015). The classical consensus nuclear localization signal (NLS) contains lysine and arginine residues (K-K/R-X-K/R), and is thus a potential target for ubiquitin ligases; however, data demonstrating that E3 ubiquitin ligases can positively regulate nuclear import via NLS ubiquitination is lacking.

Studies in macrophages treated with LPS have also shown that p65 phosphorylation at serine 536 is significantly attenuated in NKLAM^–/–^ macrophages (Lawrence et al., 2015). Phosphorylation at serine 536 is associated with NFκB transcriptional activity (Giridharan and Srinivasan, 2018). In agreement with the above studies, NKLAM^–/–^ macrophages have attenuated NFκB transcriptional activity as determined by a luciferase reporter assay, as well as lower expression of iNOS, a protein regulated by NFκB. Similarly, p65 phosphorylation in the lung is lower in NKLAM^–/–^ mice than in WT mice infected with SeV (Lawrence et al., 2019).

Collectively, these data suggest that NKLAM is involved in regulating the phosphorylation state of two immunologically important transcription factors, and in doing so, positively modulates their transcriptional activity. An attractive hypothesis is that NKLAM promotes the ubiquitin-dependent degradation of key phosphatases, resulting in maintenance of the phosphorylated state of the transcription factor, thus sustaining its activation of gene expression. Studies are ongoing to test this hypothesis.

### Regulation of Cytokine and Chemokine Expression by NKLAM

Cytokines and chemokines are immune regulators that control cell activation, migration, and differentiation. Many of the target genes of the STAT and NFκB family of transcription factors are cytokines and chemokines. This fact prompted our investigation into the regulation of cytokine and chemokine expression by NKLAM.

Early analyses of NK cells from NKLAM^–/–^ mice found that they secrete significantly less IFNγ than WT NK cells after target cell stimulation (Hoover et al., 2009). Initial *in vitro* studies demonstrated that BMDM and resident splenic macrophages from NKLAM^–/–^ mice produce significantly less IFNβ than WT macrophages following treatment with LPS (Lawrence et al., 2015). Additionally, NKLAM^–/–^ macrophages produce less IL-6, IFNγ, and MCP-1 than WT macrophages when treated with poly (I:C) (Lawrence et al., 2019). Tracheal epithelial cells isolated from NKLAM^–/–^ mice produce significantly less IL-6 and IFNγ than WT cells when infected with SeV (Lawrence et al., 2019). Importantly, this observation demonstrates that the regulation of proinflammatory cytokine production by NKLAM is not limited to immune cells. *In vivo* infection studies confirm a role for NKLAM in regulating proinflammatory cytokine expression. Both viral (SeV) and bacterial (*S. pneumoniae*) pneumonia models demonstrate that the lack of NKLAM results in significantly lower cytokine/chemokine levels in the lungs and plasma of infected mice (Lawrence and Kornbluth, 2018; Lawrence et al., 2019).

There are several chemokines among the list of cytokines tested in our animal infection studies, including MCP-1, MIP-1α, RANTES, and KC (Table 1). These chemokines serve to recruit leukocytes to sites of inflammation. In infected NKLAM^–/–^ mice, chemokine expression is significantly lower than in WT mice. This corresponds to significantly fewer leukocytes recruited into the lungs of infected mice, as determined by both flow cytometric and histologic studies (Lawrence and Kornbluth, 2018; Lawrence et al., 2019). NKLAM^–/–^ mice are not able to mount an immune response to *S. pneumoniae* and SeV comparable to WT mice.

**TABLE 1:** Proinflammatory cytokines and chemokines were measured in NKLAM^–/–^ and WT cells treated with LPS or IFNγ *in vitro* and in biological tissues and organs from NKLAM^–/–^ and WT mice exposed to live pathogens or TLR agonists *in vivo*.

**Cytokine/Chemokine:**	**Stimulus:**	**Cell Type/Tissue:**	**References**
IL-1	SeV	Lung	Lawrence et al., 2019
IL-2	SeV	Lung	Lawrence et al., 2019
IL-6	SeV, Poly (I:C)	Lung, Tracheal epithelial cells, Macrophages (BMDM)	Lawrence et al., 2019
IL-12	SeV, *S. pneumoniae*	Lung	Lawrence et al., 2019; Lawrence and Kornbluth, 2018
IL-17	SeV	Lung	Lawrence et al., 2019
IFNγ	Tumor cell stimulation, SeV, *S. pneumoniae*, Poly (I:C)	NK cells, Lung, Plasma, Macrophages (BMDM)	Hoover et al., 2009; Lawrence and Kornbluth, 2018; Lawrence et al., 2019
IFNβ	LPS, SeV	Macrophages (BMDM), Macrophages (spleen), Tracheal epithelial cells	Lawrence et al., 2015; Lawrence et al., 2019
TNFα	SeV, *S. pneumoniae*	Lung	Lawrence et al., 2019; Lawrence and Kornbluth, 2018
KC	SeV	Lung	Lawrence et al., 2019
MCP-1	SeV, *S. pneumoniae*, Poly (I:C)	Lung, Plasma, Macrophages (BMDM)	Lawrence and Kornbluth, 2018; Lawrence et al., 2019
RANTES	IFNγ, SeV	Macrophages (BMDM), Lung	Lawrence and Kornbluth, 2016; Lawrence et al., 2019
MIP-1α	SeV	Lung	Lawrence et al., 2019
MIP-1β	SeV	Lung	Lawrence et al., 2019
MIP-2	SeV	Lung	Lawrence et al., 2019
GM-CSF	SeV	Lung	Lawrence et al., 2019
G-CSF	SeV	Lung	Lawrence et al., 2019
IP-10	SeV	Lung	Lawrence et al., 2019
Eotaxin-1	SeV	Lung	Lawrence et al., 2019
AXL	SeV	Lung	Lawrence et al., 2019

Decreased cytokine and chemokine production in response to infection would suggest that NKLAM^–/–^ mice are immunocompromised. Indeed, we found this to be the case when we infected mice with a large dose of Sendai virus. Eighty percent of NKLAM^–/–^ mice died 8 days post-infection compared to only 20% of WT mice. A large, pathogenic challenge overwhelms NKLAM^–/–^ mice and leads to rapid mortality. The lack of a robust immune response by NKLAM^–/–^ mice in this instance is detrimental. However, when smaller doses of infectious agents (either SeV or *S. pneumoniae*) are given, NKLAM^–/–^ mice are afforded a slight survival benefit. This is likely due to less inflammatory leukocytes in the lungs of NKLAM^–/–^ mice, which would correspond to less host-mediated tissue destruction during inflammation.

These data suggest a model for a role of NKLAM in STAT1-mediated transcriptional activity in macrophages, in cytokine and chemokine production and in their phagosome-mediated pathogen destruction (Figure 4)^3^. Overall, these studies are defining NKLAM as a key component of the innate immune system. Further research into NKLAM substrates and potential immune signaling pathways is warranted to identify novel control points for the therapeutic modulation of inflammation.

### Links Between RBR Family Members and Immune Functions

There is very limited published data from other investigators regarding the expression/function of NKLAM. However, they collectively point to a role of NKLAM in innate immunity and response to infectious agents. One report showed that NKLAM expression is increased in chickens infected with pathogenic avian influenza virus; this increase is associated with survivability (Uchida et al., 2012). Another study documented upregulation of NKLAM expression in salmon exposed to infectious salmon anemia virus (Li et al., 2011). NKLAM levels are also significantly increased in grass carp infected with grass carp reovirus (Luo et al., 2019). The precise function of NKLAM in these infection models was not determined, but implicates NKLAM as an important component of innate immunity.

A study comparing gene expression in monocytes from adults with low versus high peak bone mass was performed to identify potential genes associated with osteoclast differentiation (Xiao et al., 2012). One of the candidate genes was NKLAM, suggesting that it plays a role in osteoporosis. NKLAM (RNF19B) gene expression was also upregulated, along with other pro-inflammatory genes, in the peripheral blood of Chinese patients with acute myocardial infarction compared to healthy controls (Su et al., 2018). Survey of the Gene Expression Omnibus (GEO)^4^ of microarray analyses identified elevation of NKLAM mRNA expression in monocytes and macrophages exposed to LPS, *Borrelia burgdorferi* (the spirochete responsible for Lyme disease), *Chlamydia pneumoniae*, *Francisella tularensis* (the causative agent for tularemia), *Porphyromonas gingivalis* (the pathogenic bacterium associated with periodontitis), *M. tuberculosis*, as well as upon infection with several adenoviruses and rhinoviruses that cause respiratory infections.

Other RBR family members have also been implicated in innate immune function. Alterations in the Parkin gene (*PARK2*) have been associated with increased susceptibility to *Salmonella typhi*, *Salmonella paratyphi* (Ali et al., 2006), and *Mycobacterium leprae* (Mira et al., 2004; Malhotra et al., 2006). Additionally, Parkin-deficient mice and *Drosophila melanogaster* are highly susceptible to infection with *M. tuberculosis* (Manzanillo et al., 2013). In a recent study, Aalto et al. (2019) found that RBR family member HOIP (HOIL-1L interacting protein) ortholog LUBEL was required for *D. melanogaster* to survive oral challenge with Gram-negative bacteria.

### Role of NKLAM in Non-Immune Functions

Hematopoietic cell activation is not the only circumstance that elicits NKLAM expression. In searching for ubiquitin ligases that may be involved in protein degradation within the endoplasmic reticulum (ER), Kaneko et al. (2016) found that HeLa cells undergoing ER stress, caused by exposure to thapsigargin or tunicamycin, upregulated NKLAM mRNA expression. In another study, it was found that MCF7 breast cancer cells upregulate NKLAM expression when exposed to oxyphenisatin acetate, a drug that was being tested as a potential cancer therapeutic (Morrison et al., 2013). This drug is known to have antiproliferative activity, but the investigators learned that treatment with this drug is also associated with autophagy, mitochondrial dysfunction, and generation of reactive oxygen species.

NKLAM mRNA expression is induced in mouse tracheal epithelial cells infected with SeV (Lawrence et al., 2019). However, these levels are extremely low compared to that seen in NK cells and macrophages. The role of non-hematopoietic cells in the phenotype we observe in NKLAM^–/–^ mice is uncertain. Reconstitution of these mice with WT bone marrow will help answer this question.

Protein ubiquitination is a critical regulatory mechanism of autophagy, controlling its initiation, execution, and termination (Chen et al., 2019). One of our recent studies suggests that NKLAM may play a role in autophagic flux. Upon induction of autophagy by treatment with rapamycin, NKLAM^–/–^ macrophages convert less LC3I to LC3II and translocate less LC3 to autophagosomes than WT macrophages. These indicators of autophagy suggest that NKLAM is associated with autophagic flux and expression of key autophagy-related proteins (Lawrence et al., 2019). Further studies are underway to analyze the role of NKLAM in autophagy.

## Future Directions

Identification of the role of the ubiquitin ligase activity of NKLAM *in vitro* and *in vivo* is of paramount importance. The derivation of an NKLAM^–/–^ NK3.3 cell line is in development to further delineate the role NKLAM plays in human NK cell effector function. This cell line, in combination with fluorescently tagged NKLAM WT and ligase-inactive constructs, will provide the ability to monitor and measure differences between WT and NKLAM^–/–^ NK cells during activation, effector function, and target cell death. Similarly, these NKLAM constructs will allow us to track NKLAM in macrophages during the process of phagocytosis and pathogen destruction and facilitate identification of NKLAM-interacting proteins during this event. Comparison of cells expressing WT and NKLAM constructs with mutations in the RBR domain (C302A) will be critical for establishing the role of ubiquitination in the function of NKLAM. Similarly, introduction of WT and catalytically inactive NKLAM into NKLAM^–/–^ mice would allow for studies of the ubiquitination function of NKLAM *in vivo*.

Experimental evidence that defines specific targets of RBR ligases is limited. The exception is RBR ligase Parkin, which has been shown to ubiquitinate dozens of proteins (Sarraf et al., 2013). The identification of NKLAM substrates would provide insight into the cellular mechanisms regulated by NKLAM-mediated ubiquitination. To this end, the use of diGly capture and MS detection methodologies followed by bioinformatic comparison of WT and NKLAM^–/–^ ubiquitomes will allow us to define specific NKLAM target proteins. Identification of NKLAM substrates, as well as the specific lysines targeted by NKLAM, will allow for the development of reagents that can be used to modulate NKLAM function in the context of innate immunity.

Several proteins that interact with NKLAM have been identified by affinity purification MS; 56 in the BioGRID database and 71 in IntAct^5^. The biological/functional validity and significance of these protein associations remains to be determined.

The prediction that intrinsically disordered domains may bind RNA or DNA provides an interesting direction for research on NKLAM. RNA-protein interactions can be divided into two categories: (1) RNA acting on the RNA binding protein (RBP) (Lunde et al., 2007) or (2) the RBP acting on the RNA (Oliveira et al., 2017). When RNA acts on the RBP, it can lead to changes in RBP function, localization, stability, or interactions. An RBP acting on the RNA can change RNA stability, processing or modification. DNA binding proteins would be localized to the nucleus and likely play a role in gene transcription, replication, repair or the regulation of those processes.

The potential for these disordered regions to interact with a variety of proteins and for these interactions to be controlled by post-translational modifications will make studying these interactions inherently difficult. NKLAM expression is tightly regulated and induced under specific activation conditions. These conditions may alter the post-translational modifications of NKLAM, thereby exposing it to different combinations of interactors (protein, DNA, and RNA). It will be necessary to not just identify which region an interacting protein binds to but also under what conditions it interacts with NKLAM, as well as differentiate between interacting proteins and ubiquitinated substrates.

Studies to date have shown that NKLAM is a unique and important ubiquitin ligase that participates in multiple regulatory pathways essential for immune function. A greater understanding of its role in controlling infection, inflammation and anti-tumor activity will hopefully lead to the development of new strategies for treating bacterial and viral infections, sepsis, autoimmunity, and cancer.

## Author Contributions

DL, PW, AC, EM, and JK contributed to the writing and editing of this review article. DL prepared the figures and table. The research performed by the Kornbluth laboratory was funded by grants obtained by JK. All authors are accountable for the content of this review article.

## Conflict of Interest

The authors declare that the research was conducted in the absence of any commercial or financial relationships that could be construed as a potential conflict of interest.

## Footnotes

1 http://alggen.lsi.upc.es/cgi-bin/promo_v3/promo/promoinit.cgi?dirDB=TF_8.3/

2 https://thebiogrid.org/

3 Adapted from Lawrence, D.W., and Kornbluth, J. E3 ubiquitin ligase NKLAM is a macrophage phagosome protein and plays a role in bacterial killing. Copyright 2012, with permission from Elsevier and Lawrence D.W., and Kornbluth, J. E3 ubiquitin ligase NKLAM ubiquitinates STAT1 and positively regulates STAT1-mediated transcriptional activity. Copyright 2016, with permission from Elsevier.

4 https://www.ncbi.nlm.nih.gov/geo/

5 https://www.ebi.ac.uk/intact/
